# The TOPLESS corepressor regulates developmental switches in the bryophyte *Physcomitrium patens* that were critical for plant terrestrialisation

**DOI:** 10.1111/tpj.16322

**Published:** 2023-06-08

**Authors:** Barry Causier, Mary McKay, Tayah Hopes, James Lloyd, Dapeng Wang, C. Jill Harrison, Brendan Davies

**Affiliations:** ^1^ Centre for Plant Sciences, Faculty of Biological Sciences University of Leeds Leeds LS2 9JT UK; ^2^ School of Molecular and Cellular Biology, Faculty of Biological Sciences University of Leeds Leeds LS2 9JT UK; ^3^ Australian Research Council Centre of Excellence in Plant Energy Biology, School of Molecular Sciences The University of Western Australia Perth WA 6009 Australia; ^4^ LeedsOmics University of Leeds Leeds LS2 9JT UK; ^5^ National Heart and Lung Institute, Imperial College London London SW3 6LY UK; ^6^ School of Biological Sciences University of Bristol 24 Tyndall Avenue Bristol BS8 1TQ UK

**Keywords:** TOPLESS, three‐dimensional growth, *Physcomitrium patens*, land plants, transcriptional corepressor

## Abstract

The plant‐specific TOPLESS (TPL) family of transcriptional corepressors is integral to multiple angiosperm developmental processes. Despite this, we know little about TPL function in other plants. To address this gap, we investigated the roles TPL plays in the bryophyte *Physcomitrium patens*, which diverged from angiosperms approximately 0.5 billion years ago. Although complete loss of PpTPL function is lethal, transgenic lines with reduced PpTPL activity revealed that PpTPLs are essential for two fundamental developmental switches in this plant: the transitions from basal photosynthetic filaments (chloronemata) to specialised foraging filaments (caulonemata) and from two‐dimensional (2D) to three‐dimensional (3D) growth. Using a transcriptomics approach, we integrated PpTPL into the regulatory network governing 3D growth and we propose that PpTPLs represent another important class of regulators that are essential for the 2D‐to‐3D developmental switch. Transcriptomics also revealed a previously unknown role for PpTPL in the regulation of flavonoids. Intriguingly, 3D growth and the formation of caulonemata were crucial innovations that facilitated the colonisation of land by plants, a major transformative event in the history of life on Earth. We conclude that TPL, which existed before the land plants, was co‐opted into new developmental pathways, enabling phytoterrestrialisation and the evolution of land plants.

## Significance Statement

TOPLESS (TPL) transcriptional corepressors are involved in multiple developmental pathways in flowering plants, but little is known about TPL function in other lineages. Using the evolutionarily distant bryophyte *Physcomitrium patens*, we show that key developmental switches that allowed plants to colonise land, namely, the switch from 2D to 3D growth and the formation of foraging tissues, require TPL. Features of TPL function therefore facilitated phytoterrestrialisation.

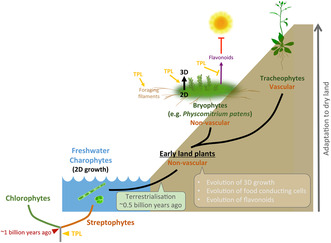

## INTRODUCTION

Organismal development relies upon multiple gene regulatory networks that respond to internal and external cues. In flowering plants, the critical transcriptional corepressor TOPLESS (TPL) family is required for numerous developmental processes and for responses to the environment (reviewed in Plant et al., 2021; Saini & Nandi, 2022). TPL proteins are recruited to specific loci through interaction with particular transcription factors (TFs), coupling them to histone deacetylases or the Mediator complex, to repress transcription of gene targets (Ito et al., 2016; Krogan et al., 2012; Leydon et al., 2021). Recruitment of TPLs to gene targets occurs directly through interaction with TFs containing a short peptide motif known as a repression domain (RD) or indirectly via RD‐containing adaptors that associate with TFs. RDs are typically five to six amino acids in length (Szemenyei et al., 2008; Kagale & Rozwadowski, 2011; Causier, Ashworth, et al., 2012) and may be acquired by any TF through a small number of amino acid substitutions, delivering new functionality to these proteins through recruitment of the TPL regulator, consequently integrating TPL into new developmental pathways to drive innovation (Plant et al., 2021). Although the role of TPL in hormone signalling, flowering, leaf development, meristem maintenance, pathogen resistance and many other processes has been extensively studied in angiosperms (reviewed in Plant et al., 2021), its evolutionary history remains unresolved. Here we aimed to address these outstanding questions and determine the developmental pathways into which TPL was recruited early in land plant evolution.

The bryophyte *Physcomitrium patens* (formerly *Physcomitrella patens*; Medina et al., 2019), which diverged from angiosperms approximately 0.5 billion years ago, has features of ancestral land plants and has been key to our understanding of land plant evolution (Rensing et al., 2020). This plant comprises a two‐dimensional (2D) spreading mat of basal assimilatory filaments (chloronemata) and foraging filaments (caulonemata) that comprise food‐conducting cells necessary for resource acquisition in dry habitats (reviewed in Ligrone et al., 2012). Once a filamentous plant is established, a proportion of caulonemal cells divide asymmetrically to produce three‐dimensional (3D) upright leafy shoots, known as gametophores, which bear the reproductive organs (Harrison et al., 2009; Rensing et al., 2020; Thelander et al., 2018). The genetic pathways regulating the chloronemata‐to‐caulonemata transition and the 2D‐to‐3D growth transition are well characterised, involving multiple hormone pathways, TFs and their downstream targets (Aoyama et al., 2012; Goss et al., 2012; Jaeger & Moody, 2021; Moody et al., 2018; Moody et al., 2021; Nemec‐Venza et al., 2022; Perroud et al., 2014; Pires et al., 2013; Tam et al., 2015; Whitewoods et al., 2018; Whitewoods et al., 2020).

We disrupted *P. patens* TPL (PpTPL) protein activity and show that these corepressors are required for critical 2D‐to‐3D and chloronemata‐to‐caulonemata developmental switches. Using a transcriptomic approach, we link phenotypes to changes in gene expression, showing that PpTPLs have been independently recruited into diverse gene regulatory pathways. We also show that PpTPL regulates the synthesis of flavonoids, compounds that protect plants against harmful solar radiation (Ferreyra et al., 2021). Colonisation of new areas and volumes of space and resistance to detrimental environmental conditions were pivotal in plants' colonisation of land, suggesting that TPL facilitated key evolutionary events in Earth's history.

## RESULTS

### PpTPL activity is required for viability

**Figure 1:**
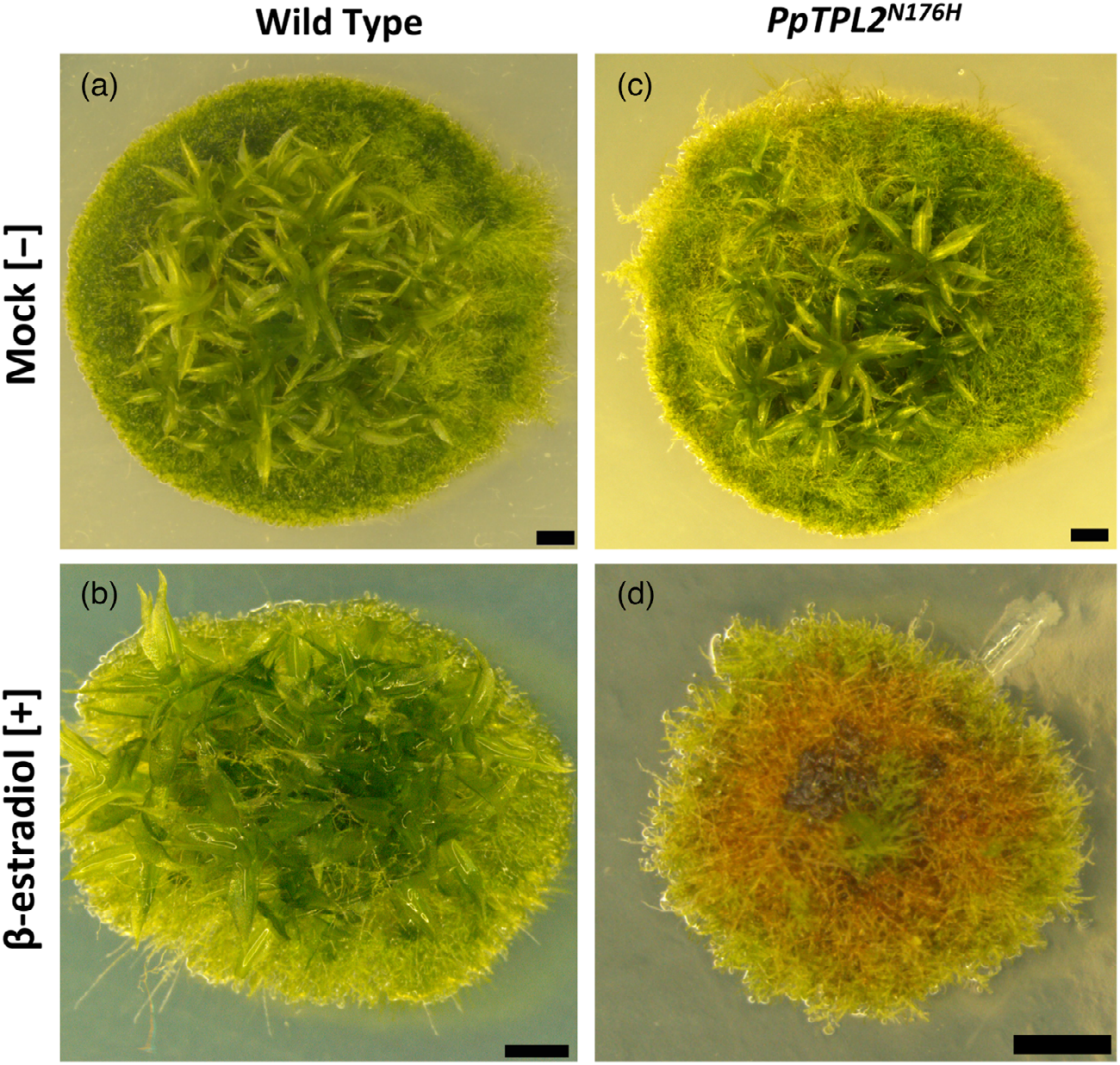
Reduced TPL activity disrupts *P. patens* development. (a) Typical mock‐treated wild‐type plant (WT[−]). Filamentous growth can be seen at the edge of the plant, with leafy shoots (gametophores) at the centre. (b) Typical wild‐type plant treated with 1 μm β‐estradiol (WT[+]). (c) Typical mock‐treated *PpTPL2* ^*N176H*^ transgenic plant (*PpTPL2* ^*N176H*^[−]). Note that plants in panels (b) and (c) are phenotypically similar to the wild‐type plant in (a). (d) *PpTPL2* ^*N176H*^ plant treated with 1 μm β‐estradiol (*PpTPL2* ^*N176H*^[+]), showing loss of mature gametophores and red/brown hyperpigmentation. All plants are approximately 6 weeks old. Scale bars = 1 mm.

The *P. patens* genome contains two genes predicted to encode TPL‐related corepressors, named *PpTPL1* (Pp3c15_9880V3) and *PpTPL2* (Pp3c9_21250V3) (Causier, Lloyd, et al., 2012), which are both broadly expressed in the same tissues throughout *P. patens* development (Figure S1). To determine the biological functions of these proteins, we first used gene targeting to delete each gene from the genome. Verified *ΔPptpl1* and *ΔPptpl2* single knockouts were indistinguishable from wild‐type plants (Figure S2), indicating that, as in Arabidopsis (Long et al., 2006), *P. patens TPL* genes function redundantly, consistent with the overlapping expression patterns of both *PpTPL* genes (Figure S1). We then attempted to generate double knockouts in each single mutant background, but regardless of the background used as a starting point, we were unable to isolate *ΔPptpl1 ΔPptpl2* double mutants, indicating that TPL activity is essential for viability. We therefore adopted alternative strategies to investigate PpTPL function. We first constitutively expressed a dominant negative version of *PpTPL2* (*PpTPL2* ^*N176H*^), carrying the same point mutation found in the dominant Arabidopsis *tpl‐1* mutant (Long et al., 2006), in wild‐type Arabidopsis plants. This led plants to develop *tpl‐1*‐like phenotypes (Figure S2), indicating that expression of *PpTPL2* ^*N176H*^ in *P. patens* should be sufficient to disrupt the function of PpTPL1 and PpTPL2 proteins. Next we generated a conditional *PpTPL2* ^*N176H*^ cassette with β‐estradiol‐inducible expression (Figure S2) to generate *P. patens* PpTPL dominant negative mutants. When grown on induction media, *PpTPL2* ^*N176H*^ transgenics (*PpTPL2* ^*N176H*^[+]) showed clear phenotypic differences from control *P. patens* plants (Figure 1). Subsequent analyses of TPL function in *P. patens* were based on two independent *PpTPL2* ^*N176H*^ lines, which were viable and behaved similarly in all experiments.

Failure to isolate *ΔPptpl1 ΔPptpl2* double mutants may potentially reflect an inability of protoplasts (prepared during the transformation protocol) to regenerate, rather than an issue with viability in loss‐of‐function mutants. However, protoplasts prepared from *PpTPL2* ^*N176H*^[+] were able to regenerate, even in the presence of β‐estradiol (Figure S2), suggesting that while PpTPL activity may not be necessary for protoplast regeneration, it is required for viability of *P. patens* plants. As a caveat, we cannot exclude the possibility that β‐estradiol does not induce gene expression in *P. patens* protoplasts, although this compound is an effective inducer of transcription in Arabidopsis mesophyll and root protoplasts (Dory et al., 2018; Schlücking et al., 2013).

### TPL is required for the 2D‐to‐3D developmental switch in *P. patens*

**Figure 2:**
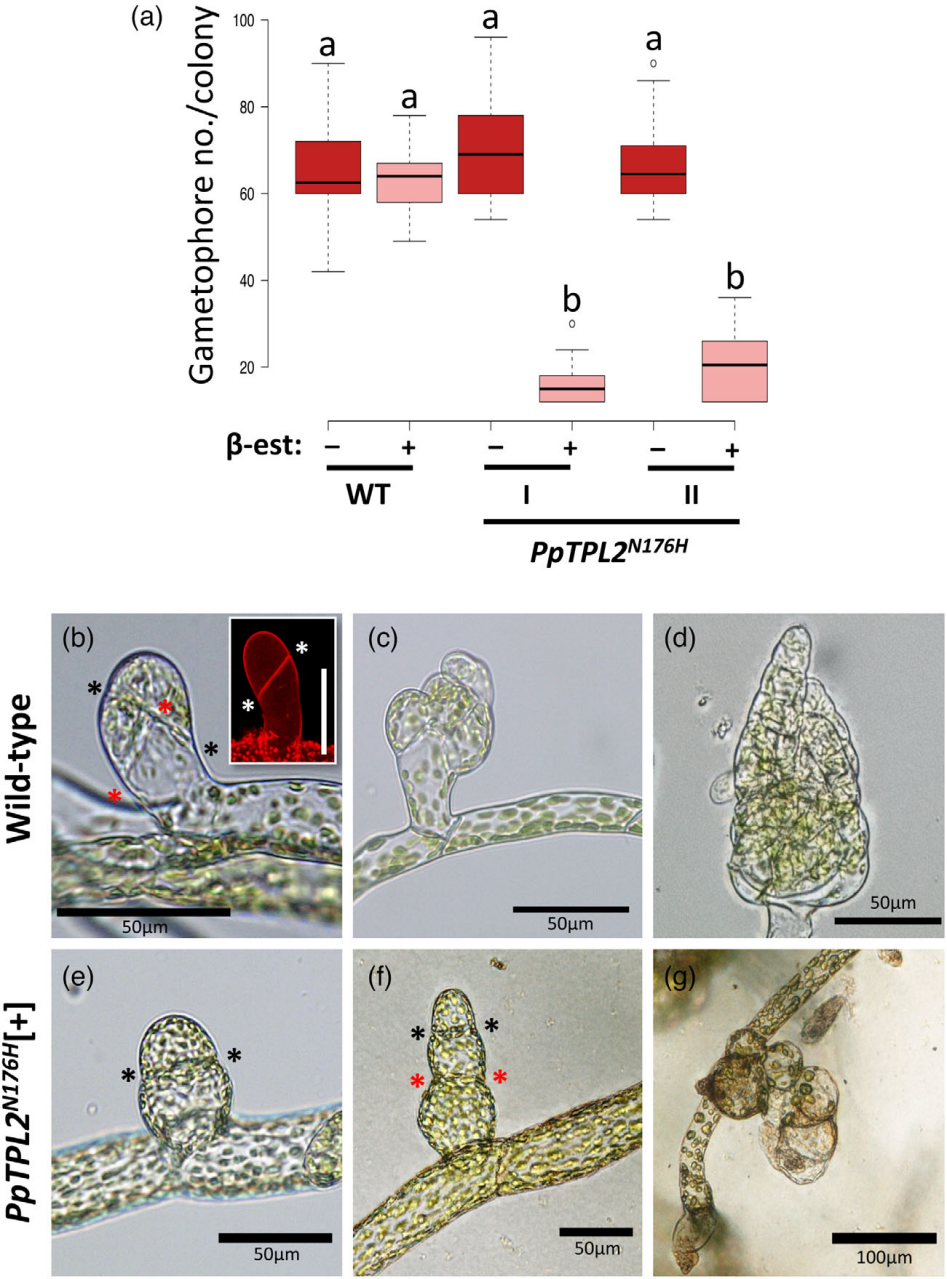
PpTPL is required for gametophore development. (a) Box and whisker plot for numbers of gametophore structures with at least one leaf counted in dissected 5‐week‐old colonies (*n* > 10). Centre lines show the medians; box limits indicate the 25th and 75th percentiles as determined by R software; whiskers extend 1.5 times the interquartile range from the 25th and 75th percentiles; outliers are represented by dots. Two independent *PpTPL2* ^*N176H*^ lines (I and II) were measured. [−] indicates mock‐treated control plants, [+] indicates plants grown on media supplemented with 1 μm β‐estradiol. Boxes with different letters are significantly different from one another (Tukey HSD inference; *P* < 0.01). (b) First bud cell divisions in wild‐type plants, with the first oblique division shown in the inset and highlighted with asterisks (scale bar = 50 μm). Subsequent asymmetric cell divisions are marked by asterisks in the main panel. (c, d) Later stages of gametophore formation. (e) The first cell division (marked by asterisks) in *PpTPL2* ^*N176H*^ plants treated with 1 μm β‐estradiol (*PpTPL2* ^*N176H*^[+]), which is misoriented relative to wild type (b). (f, g) Subsequent cell divisions are also misoriented in *PpTPL2* ^*N176H*^[+] plants, leading to the production of malformed callus‐like structures in place of normal gametophores (compare panels (d) and (g)).

The most striking phenotype of *PpTPL2* ^*N176H*^[+] plants was a significant reduction in the formation of gametophores relative to control plants (Figures 1 and 2a), indicating defects in the switch from 2D to 3D growth. A number of *P. patens* mutants have been described in which gametophore development is suppressed due to disruption of cell divisions early in 3D growth (reviewed in Moody, 2019). To understand how the TPL corepressors promote 3D growth, we examined those critical early cell divisions in our *PpTPL2* ^*N176H*^[+] lines. In wild‐type plants, bud swelling and an initial oblique cell division mark the transition to 3D growth (see Figure 2b), and a series of characteristic asymmetric cell divisions generate highly organised gametophore structures (Harrison et al., 2009; Tang et al., 2020; Figure 2c,d). However, the earliest cell divisions in the *PpTPL2* ^*N176H*^[+] plants were misplaced (Figure 2e,f), resulting in malformed buds that give rise to aborted, callus‐like structures (Figure 2g). Treatment of *P. patens* plants with β‐estradiol has no effect on development (Kubo et al., 2013) or on early cell divisions during the transition to 3D growth (Figure S2). To verify that formation of gametophores requires TPL activity, we also generated β‐estradiol‐inducible RNA interference (RNAi) lines to deplete *PpTPL1* expression in *ΔPptpl2* single mutants (herein referred to as *ΔPptpl2 PpTPL1* ^*RNAi*^) or *PpTPL2* expression in *ΔPptpl1* single mutants (*ΔPptpl1 PpTPL2* ^*RNAi*^) (Figure S3). As in *PpTPL2* ^*N176H*^[+] lines, gametophore development was significantly inhibited in β‐estradiol‐treated RNAi plants, with similar defects in early cell divisions (Figure S3). In summary, our data reveal that TPL activity promotes the 2D‐to‐3D growth switch by correctly orientating cell divisions in gametophore initials.

### Genes involved in 3D growth are differentially expressed in *PpTPL2* ^*N176H*^ [+] plants

A number of genes have been shown to be required for the switch to 3D growth in *P. patens* (reviewed in Moody, 2019). To understand the position of PpTPL in the gene regulatory network governing 3D growth, we compared the transcriptomes of *PpTPL2* ^*N176H*^[+] and control plants (mock‐treated wild type, β‐estradiol‐treated wild type and mock‐treated *PpTPL2* ^*N176H*^[−]) to generate a high‐confidence dataset that identified 1901 differentially expressed genes (DEGs) (>2‐fold change in expression; *P*_adj_ <0.05, Figure S4). Statistics of generated reads, mapping data and DEG analyses are presented in Tables [Link], [Link] and Figure S4.

**Figure 3:**
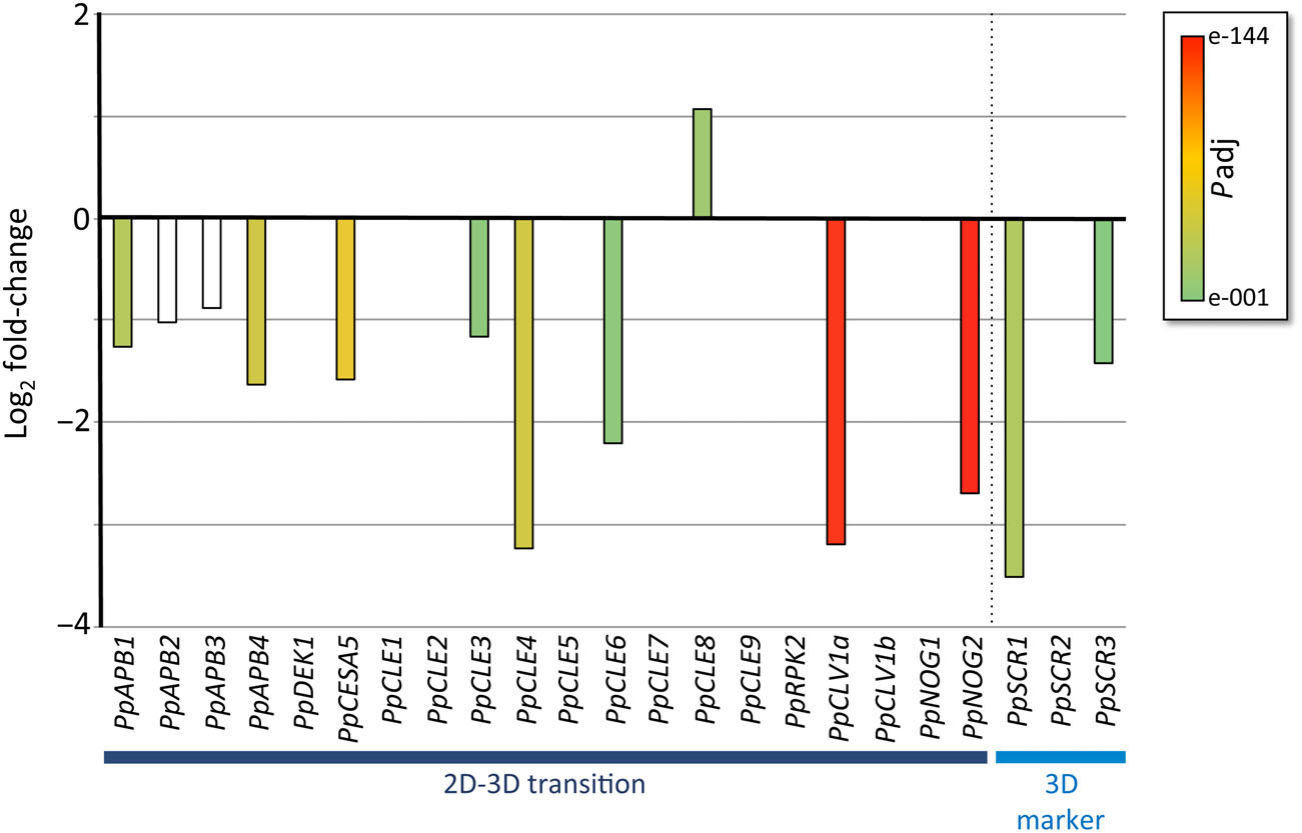
Genes involved in the switch to 3D growth show altered expression in *PpTPL* dominant negative plants. Expression profiles of candidate genes linked to 3D growth in *PpTPL2* ^*N176H*^[+] plants relative to controls. Bar colours represent adjusted *P*‐values (*P*_adj_) as a measure of statistical significance (see key inset). Unfilled bars (*PpAPB2* and *PpAPB3*) are not significant.

Among the genes already shown to be necessary for 3D growth in *P. patens*, *AINTEGUMENTA*, *PLETHORA* and *BABY‐BOOM*‐related genes (*PpAPB1*–*4*), *NO GAMETOPHORES 2 (PpNOG2)*, *PpCLAVATA1a* (*PpCLV1a*) and *CELLULOSE SYNTHASE 5* (*PpCESA5*) (Aoyama et al., 2012; Goss et al., 2012; Moody et al., 2021; Whitewoods et al., 2018; Whitewoods et al., 2020) were significantly downregulated in *PpTPL2* ^*N176H*^[+] plants, whilst expression of other 3D growth genes, including *DEFECTIVE KERNEL1* (*PpDEK1*), *PpNOG1*, *PpCLV1b*, *PpRPK2*, *PpCLAVATA3‐like 1* (*PpCLE1*), *PpCLE2* and *PpCLE7* (Moody et al., 2018; Perroud et al., 2014; Whitewoods et al., 2018; Whitewoods et al., 2020), was unchanged (Figure 3, Table S3). Therefore, of all the genes currently known to be required for 3D growth, half show reduced expression in *PpTPL2* ^*N176H*^[+] plants. In addition, *SCARECROW1* (*PpSCR1*) and *PpSCR3*, which are markers of 3D growth in *P. patens* (Moody et al., 2021), are also significantly downregulated in *PpTPL2* ^*N176H*^[+] plants (Figure 3).

To confirm that misregulation of 3D growth genes in *PpTPL2* ^*N176H*^[+] plants is due to loss of PpTPL activity, we compared DEGs identified in *PpTPL2* ^*N176H*^[+] with those found in transcriptomics data obtained for the *ΔPptpl2 PpTPL1* ^*RNAi*^[+] line (compared to the same controls as the *PpTPL2* ^*N176H*^[+] samples), which represents an orthogonal dataset to validate *PpTPL2* ^*N176H*^[+] DEGs on a global scale, without bias. Consistent with this, we identified a significant overlap in up‐ and downregulated genes, and in Gene Ontology (GO) term enrichment, between the *PpTPL2* ^*N176H*^[+] and *ΔPptpl2 PpTPL1* ^*RNAi*^[+] datasets (Figure S5). Such comparisons revealed that, as in *PpTPL2* ^*N176H*^[+], *PpAPB1*, *PpAPB4*, *PpNOG2*, *PpCLV1a*, *PpCESA5* and *PpSCR1* were all downregulated in *ΔPptpl2 PpTPL1* ^*RNAi*^[+] plants (Table S3). The transcriptomic data show that PpTPL operates upstream of genes that have critical roles in gametophore initiation and provide a genetic rationale for the failure of plants with defective PpTPL function to initiate the formation of normal 3D buds and form mature gametophores.

### Transcriptomics indicate a role for PpTPLs in the regulation of flavonoid biosynthesis

**Figure 4:**
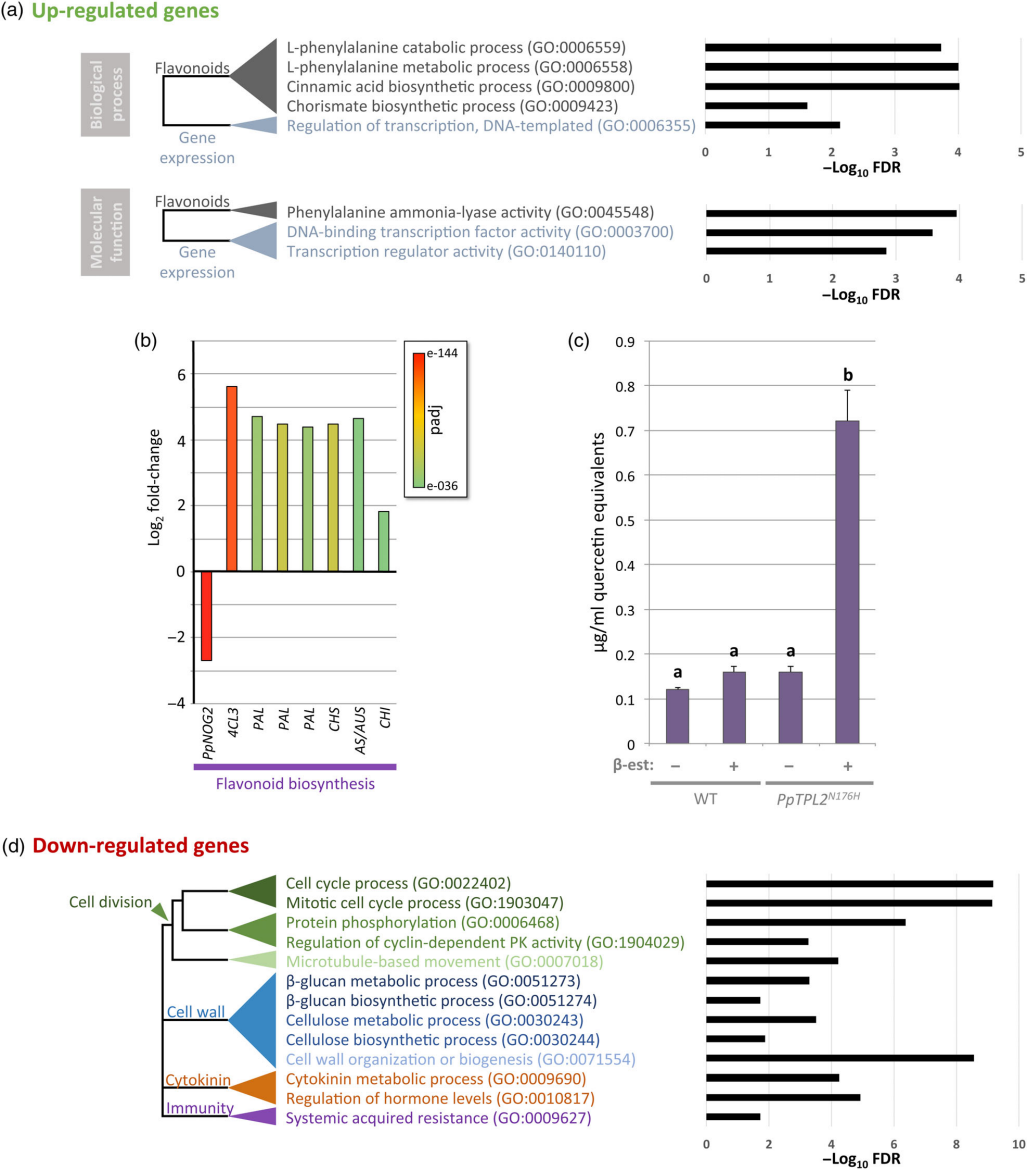
The expression of genes involved in diverse biological processes is altered in *PpTPL* dominant negative plants. (a) Enrichment of Gene Ontology (GO) terms associated with genes upregulated in *PpTPL2* ^*N176H*^[+] plants. GO terms associated with biological processes (top) or molecular functions (bottom) are shown. The dendrograms on the left show the relationships between GO terms. The charts on the right shows the log_10_ (false discovery rate [FDR]) value. (b) Expression profiles of candidate genes linked to flavonoid biosynthesis in *PpTPL2* ^*N176H*^[+] plants relative to controls. Bar colours represent adjusted *P*‐values (*P*_adj_) as a measure of statistical significance (see key inset). (c) Quantification of total flavonoid content in extracts from wild‐type (WT) and *PpTPL2* ^*N176H*^ plants, grown in the presence [+] or absence [−] of 1 μm β‐estradiol. Different letters indicate significant differences (Tukey HSD inference; *P* < 0.01). (d) GO terms enriched for genes downregulated in *PpTPL2* ^*N176H*^[+] plants. The dendrogram to the left shows the relationships between GO terms. The chart on the right shows the log_10_(FDR) value.

To determine the global change in gene expression during the 2D‐to‐3D transition and to obtain an overview of other biological pathways in which PpTPLs may function, we performed a GO term enrichment analysis separately for the up‐ and downregulated DEGs (Figure S4). Analysis of the 675 genes upregulated in *PpTPL2* ^*N176H*^[+] plants revealed an overrepresentation of factors associated with flavonoid biosynthesis (Figure 4a, Table S4). Consistent with this, genes encoding enzymes positioned at various points in the flavonoid biosynthesis pathway (see Davies et al., 2020), such as 4‐coumarate:CoA ligase 3 (4CL3), chalcone synthase (CHS), aurone synthase (AS) and phenylalanine ammonia‐lyase (PAL), were among the most significantly upregulated genes in *PpTPL2* ^*N176H*^[+] plants (Figure 4b and Table S3), suggesting that flavonoids may accumulate in these plants. To test this, we quantified flavonoids and found that filaments of *PpTPL2* ^*N176H*^[+] plants had a total flavonoid content approximately 4.5‐fold higher than control plants (WT[−], WT[+] and *PpTPL2* ^*N176H*^[−] plants) (Figure 4c). Together, our transcriptomic and biochemical data suggest that PpTPLs repress flavonoid production. Flavonoids are an important group of plant secondary metabolites and pigments, which may have acted as a sunscreen in early land plants (reviewed in Davies et al., 2020). Accumulation of flavonoids in *PpTPL2* ^*N176H*^[+] plants also explains the striking red/brown hyperpigmentation observed in these lines (Figure 1).

Unexpectedly for plants with loss of a transcriptional corepressor, the expression of almost twice as many genes was downregulated in *PpTPL2* ^*N176H*^[+] plants than upregulated (Figure S4). While this might be partly explained by the presence of direct and indirect targets within the datasets, intriguingly, it may also indicate that, as has been shown for repressors in other eukaryotes (Baymaz et al., 2015; Reynolds et al., 2013), TPLs could have critical roles in both repression and activation of gene expression. Analysis of these genes downregulated in *PpTPL2* ^*N176H*^[+] plants revealed enrichment for GO terms related to diverse biological processes, including cytokinin (CK) metabolism, cell division and cell wall formation (Figure 4d; and see Table S3), highlighting the potential array of molecular targets and biological pathways in *P. patens* that involve TPL activity.

### PpTPLs are required for the developmental switch from assimilatory to foraging filament identity

**Figure 5:**
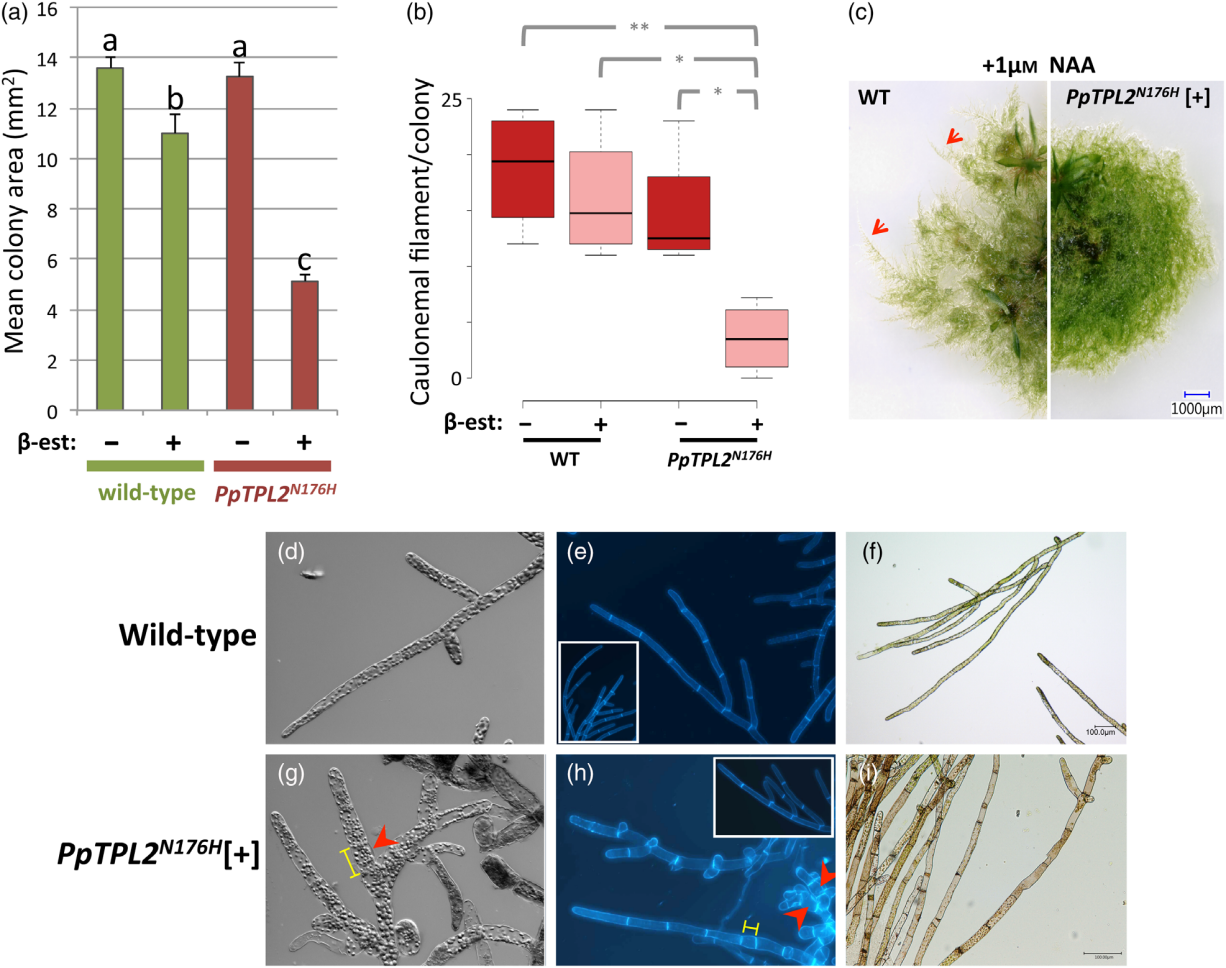
PpTPL is necessary for the chloronema‐to‐caulonema developmental switch. (a) Mean colony area (±SEM; *n* ≤ 12) for wild‐type and *PpTPL2* ^*N176H*^ plants, grown with [+] or without [−] 1 μm β‐estradiol (β‐est). Different letters indicate significant differences (Tukey HSD inference; *P* < 0.01). (b) Box and whisker plot for average number of caulonemal filaments produced per plant (*n* = 4) under dark‐grown conditions. Centre lines show the medians; box limits indicate the 25th and 75th percentiles as determined by R software; whiskers extend 1.5 times the interquartile range from the 25th and 75th percentiles. Statistically significant differences between samples are shown (Tukey HSD inference; **P < 0.01, *P < 0.05). (c) *Physcomitrium patens* plants grown in the presence of 1 μm exogenous auxin (NAA). Wild‐type plant (left) and *PpTPL2* ^*N176H*^ plant treated with 1 μm β‐estradiol (right; *PpTPL2* ^*N176H*^[+]). In the presence of NAA, WT plants produce caulonemal filaments (red arrows), giving a feathery colony edge, whilst *PpTPL2* ^*N176H*^[+] plants do not. (d–f) Typical chloronemal filaments produced by wild‐type plants under control conditions. (g–i) Protonemal filaments produced by *PpTPL2* ^*N176H*^ plants treated with 1 μm β‐estradiol (*PpTPL2* ^*N176H*^[+]). Note that some cells are swollen (red arrow heads) and/or shorter (yellow bars) than in WT filaments (g and h). Also note the red/brown pigmentation and the heterogeneous nature of some filaments (i). For example, the filaments in (i) have properties of both caulonemata (red/brown pigment not found in chloronemata) and chloronemata (perpendicular cell walls, rather than the oblique cell walls characteristic of caulonemata). Micrographs in panels (d) and (g) are at the same magnification. Micrographs in panels (e) and (h) are at the same magnification. Scale bars in (f) and (i) = 1 μm.

Further analysis of *PpTPL2* ^*N176H*^[+] plants showed that, in addition to their failure to switch from 2D to 3D growth, they also had a significantly reduced surface area compared to control plants (Figure 5a), suggesting possible defects in filament formation. Examination of filament morphologies in *PpTPL2* ^*N176H*^[+] plants revealed a variety of phenotypes, such as filaments with mixed identities, sharing features of both chloronemata (perpendicular cell walls) and caulonemata (pigmentation) (Figure 5; Figure S6), and formation of abnormally short and/or swollen cells (compare Figure 5d,e with Figure 5g,h, respectively, and see Figure S6). Reduced plant spread is indicative of problems with formation of caulonemata, which are faster growing and longer than chloronemata (Tam et al., 2015). To assess caulonemal development, we counted the number of caulonemal filaments produced by plants grown in the dark (Hoffmann et al., 2014). We found that formation of these foraging filaments was significantly suppressed in both *PpTPL2* ^*N176H*^[+] and *ΔPptpl2 PpTPL1* ^*RNAi*^[+] plants, relative to controls (Figure 5b; Figure S6), confirming a role for TPL in the developmental switch from chloronemal to caulonemal identity in *P. patens*. Formation of caulonemata is induced by auxin, so next we treated plants with exogenous auxin and looked for induction of caulonemal filaments. In WT, 1 μm NAA is sufficient to induce caulonemata (Figure 5c). However, when treated with 1 μm NAA no caulonemal filaments were produced by *PpTPL2* ^*N176H*^[+] (Figure 5c) or *ΔPptpl2 PpTPL1* ^*RNAi*^[+] plants (Figure S6). Even at high NAA concentrations (e.g. 10 μm NAA), caulonemal production was still suppressed in *PpTPL2* ^*N176H*^[+] plants (Figure S6), indicating that phenotypes may be due to disrupted auxin perception or transport, rather than altered levels of endogenous auxin.

## DISCUSSION

### TPL is essential for developmental switches in *P. patens*

In angiosperms, TPL activity is integral to numerous developmental programmes and responses to the environment (Plant et al., 2021). As a consequence, loss of TPL activity results in dramatic outcomes, ranging from the profound developmental defects of the Arabidopsis *tpl‐1* mutant (Long et al., 2002) to lethality, which we report here for *P. patens*. The severity of these phenotypes presents an obstacle to discovering the full range of roles played by this family of corepressors.

Here, using non‐lethal dominant negative and RNAi transgenic lines, we show that TPLs are required for two major developmental switches in *P. patens*. The basal tissue of a *P. patens* plant is the photosynthetic chloronemal filament, which constitutes the first tissue type to develop from *P. patens* germinating spores (reviewed in Rensing et al., 2020). Chloronemal cells can self‐renew or may differentiate to form foraging caulonemal cells (reviewed in Jaeger & Moody, 2021). Caulonemata are specialised filaments made up of food‐conducting cells (FCCs) with features similar to tracheophyte sieve elements (reviewed in Ligrone et al., 2012). The switch to caulonemal growth is regulated by resource availability, but is also controlled by a balance between auxin, which promotes caulonemata, and CK, which inhibits caulonemal growth (reviewed in Kofuji & Hasebe, 2014). The regulatory pathways necessary for the transition from basal chloronemata to specialised caulonemata are beginning to be unravelled (Menand et al., 2007; Tam et al., 2015; reviewed in Jaeger & Moody, 2021) and here we show that PpTPLs are required for this developmental switch (Figure 5b; Figure S6).

Caulonemal cells give rise to side branch initials that can either develop as additional filaments to maintain growth in 2D or switch to the 3D growth phase (Cove & Knight, 1993). The transition to 3D growth is significantly inhibited in both *PpTPL2* ^*N176H*^[+] and RNAi plants, indicating that TPL is also critical for this developmental switch. The reduction in the transition to 3D growth cannot be solely explained by the suppression of caulonemata in plants with reduced PpTPL function, since the buds that do form are characterised by misoriented early cell divisions, leading to the formation of callus‐like structures (Figure 2; Figure S3). In summary, TPL is independently required for both the switch from chloronemal to caulonemal growth and the transition from 2D to 3D growth.

### TPL and auxin responses in *P. patens*

In angiosperms, TPL proteins play roles in multiple hormone signalling pathways (reviewed in Plant et al., 2021). One of the earliest roles identified for TPL was as a critical component of the auxin signalling mechanism, interacting with AUX/IAA proteins to repress auxin‐responsive genes in the absence of auxin (Szemenyei et al., 2008). In bryophytes, *aux/iaa* or *tpl* mutants of *Marchantia* and *aux/iaaΔ* null mutants of *P. patens* show constitutive auxin phenotypes (Flores‐Sandoval et al., 2015; Lavy et al., 2016), consistent with a conserved role for these proteins in auxin signalling throughout the land plants.

There are striking similarities between the phenotypes of the *aux/iaaΔ* loss‐of‐function mutants (Lavy et al., 2016) and the *PpTPL2* ^*N176H*^[+] plants described here, namely, formation of disorganised and hyperpigmented protonemal filaments (Figure 5). However, while there are statistically significant overlaps in transcripts differentially regulated in both mutants that may explain the phenotypic similarities (Figure S7), several of the auxin‐responsive genes upregulated in *aux/iaaΔ* are downregulated in *PpTPL2* ^*N176H*^[+] (Table S3). One possibility is that TPL may act to repress pathways that antagonise auxin responses, explaining the strong suppression of auxin‐induced caulonemata in *PpTPL2* ^*N176H*^[+] (Figure 5; Figure S6), but not *aux/iaaΔ* plants (Lavy et al., 2016).

CKs are potent inhibitors of caulonemata formation (Ashton et al., 1979; Kofuji & Hasebe, 2014). Cytokinin oxidase genes (CKX), which control intracellular CK levels, are downregulated in *PpTPL2* ^*N176H*^[+]. This may partially explain why *PpTPL2* ^*N176H*^[+] plants share some similarity with wild‐type plants grown on exogenous CK (Figure 5; Figures S3 and S6), including pigmented filaments made up of short, swollen cells, failure to initiate caulonemata and undifferentiated callus‐like buds (Ashton et al., 1979; Thelander et al., 2005). However, despite these constitutive CK‐like phenotypes, we do not observe the expected upregulation of CK‐responsive genes in these plants (Figure S7; Table S3).

Auxin responses are also disrupted by flavonoids, which can inhibit auxin export (Brown et al., 2001). For example, in the *ppnog2* mutant, which shows defects in the early cell divisions of 3D growth similar to *PpTPL2* ^*N176H*^[+], it is hypothesised that loss of this shikimate *O*‐hydroxycinnamoyl‐transferase‐encoding gene alters metabolic flux, resulting in enhanced levels of flavonoids (Moody et al., 2021). *PpNOG2* is significantly downregulated in *PpTPL2* ^*N176H*^[+] plants. In addition, genes encoding different flavonoid biosynthesis enzymes are significantly upregulated in these plants (Table S3). Together this results in the accumulation of flavonoids in *PpTPL2* ^*N176H*^[+] plants (Figure 4c), potentially resulting in defects in auxin‐responsive developmental processes such as induction of caulonemata and 3D growth. However, *PpNOG2*, various flavonoid biosynthesis genes and *CKX* genes are also differentially regulated in *aux/iaaΔ*, in a similar manner to that seen for *PpTPL2* ^*N176H*^[+] (Table S3), supporting the similarities in the phenotypes of these mutants, but not the differences in constitutive auxin responses.

While many DEGs are shared between the *aux/iaaΔ* and *PpTPL2* ^*N176H*^[+] plants, there are also large numbers of genes uniquely misregulated in each mutant (Figure S7), suggesting that TPL and AUX/IAAs have additional, independent roles. This is not a surprise given that TPL predates the auxin signalling pathway (see Plant et al., 2021). GO analysis of genes only downregulated in *PpTPL2* ^*N176H*^[+] reveals enrichment of terms associated with two major biological processes: cell wall biogenesis and the cell cycle (Figure S7). Cell wall modifications are necessary to support growth in 3D (de Vries & Archibald, 2018). Within the cell wall biogenesis class are genes encoding cellulose synthase genes, notably *PpCESA5*, mutations in which cause major defects in 3D growth (Goss et al., 2012). Many genes linked to the cell cycle and setting of cell division sites are also uniquely downregulated in *PpTPL2* ^*N176H*^[+] (Table S3), including genes belonging to the *PpTPX2* family that are required for the orientation of the cell division plane early in 3D development (Kozgunova et al., 2022). Changes in the expression of specific genes, or general misregulation of the cell cycle, may conceivably disrupt the reprogramming of new cell identities (Ishikawa et al., 2011), contributing to the defects in both the transition to 3D growth and the formation of caulonemata in *PpTPL2* ^*N176H*^[+] plants.

Although the individual contributions are still to be determined, it seems likely that *PpTPL2* ^*N176H*^[+] phenotypes are the result of increased flavonoid levels and reduced *CKX*, cell wall and cell cycle gene expression, showing that, as in Arabidopsis, PpTPLs are associated with multiple developmental pathways.

### Where does TPL fit into the 3D growth gene regulatory network?

**Figure 6:**
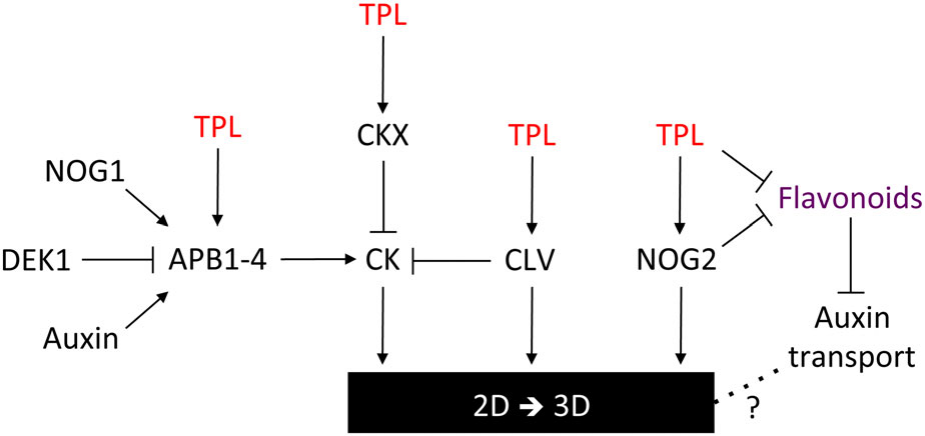
Model for TPL function in the gene regulatory network that controls 3D growth in *P. patens*. During the initial stages of 3D bud initiation, TPL operates upstream of APB1–4, CKX, CLV signalling and NOG2, all of which are required for normal transition to 3D growth. Arrows and T‐bars indicate gene activation and repression, respectively. A dotted line indicates that any functional interaction remains to be resolved.

The evolution of 3D growth occurred early in the land plant lineage (reviewed in Harrison, 2017). A number of genes have now been identified that are required for 3D growth in *P. patens* (reviewed in Moody, 2019), and we propose that PpTPLs represent another important class of regulators that are essential for the 2D‐to‐3D developmental switch. Although further work is required to understand how these corepressors fit into a fully integrated 3D growth gene regulatory network, many genes shown to be important for the transition from 2D to 3D growth show altered expression in *PpTPL2* ^*N176H*^[+]. For example, all four *PpAPB* genes, which are master regulators of this process (Aoyama et al., 2012), are downregulated in *PpTPL2* ^*N176H*^[+] plants (Figure 3). Similarly, we identified several genes encoding components of the CLV pathway that are downregulated in *P. patens* plants with disrupted TPL function (Figure 3). Similar to *PpTPL2* ^*N176H*^[+] and the RNAi lines (Figure 2; Figure S3), CLV mutants show early defects in bud formation (Whitewoods et al., 2018; Whitewoods et al., 2020). Finally, we identified *PpNOG2* as another significantly downregulated gene in our transcriptomic dataset (Figure 3), which may also explain perturbations in 3D growth in these mutants. Taken in the round, our data suggest that PpTPLs have been independently recruited into multiple points of the gene regulatory network controlling the transition to 3D growth (Figure 6).

### On the evolution of TPL function

TPL is plant‐specific, and we have previously shown that it is ancient, probably existing near the origin of the green lineage (Plant et al., 2021). Currently, our understanding of TPL function, evolution and mode of action is based on studies in angiosperms, revealing how this family of transcriptional corepressors has become essential to multiple aspects of plant development and responses to the environment (reviewed in Plant et al., 2021). Despite 20 years of intensive research, we know little about TPL function outside of the flowering plants. Angiosperms and bryophytes diverged approximately 500 million years ago (Morris et al., 2018) and have since followed independent evolutionary trajectories. Some functions of TPL appear to predate this split. These include interaction with AUX/IAA proteins to control auxin signalling across the land plant lineage (Causier, Lloyd, et al., 2012; Flores‐Sandoval et al., 2015; Szemenyei et al., 2008), regulation of *CKX* gene expression in *P. patens* (Table S3) and in angiosperms (He et al., 2021; Parker et al., 2021) and functions in CLV signalling, which is required to correctly orient cell division planes during 3D growth in *P. patens* and angiosperms (Whitewoods et al., 2018; Whitewoods et al., 2020). Our transcriptomic data reveal that PpTPLs are positioned upstream of CLV signalling (Figure 6), and data from rice (*Oryza sativa*) show that this is also the case in angiosperms (Suzuki et al., 2019), suggesting that the involvement of TPL in CLV signalling originated with the land plants, coincident with the evolution of 3D growth.

An important question is, how did TPL become so vital for plant life? We argue that this was enabled by the ready recruitment of TPL proteins into new pathways. TPL is recruited by TFs containing a surprisingly broad range of short RD sequences (Kieffer et al., 2006; Causier, Ashworth, et al., 2012; Causier, Lloyd, et al., 2012; Zhang et al., 2014; Pi et al., 2015; Dolzblasz et al., 2016; Graeff et al., 2016; Goralogia et al., 2017; Uhrig et al., 2017). RDs are examples of short linear motifs (SLiMs), which are evolutionarily plastic functional domains that promote interactions with other proteins (Davey et al., 2015; Edwards & Palopoli, 2015). SLiMs have been proposed to enable novel protein–protein interactions and, thereby, facilitate the evolution of phenotypic novelties. We speculate that RDs may be readily acquired by any TF through a small number of amino acid substitutions in the TF or an adaptor protein, allowing TPL to reshape existing gene regulatory networks and drive innovation.

### TPL and the emergence of land plants

The successful colonisation of land by plants was one of the most important evolutionary events for life on Earth. Land plants evolved once from an aquatic charophyte algal ancestor with the transition to land requiring numerous adaptations to adjust to the radical challenges presented by a novel, arid environment (reviewed in de Vries & Archibald, 2018; Moody, 2020). It is unclear how the concerted range of phenotypic and metabolic changes required for life on land were enabled, but they were likely the product of newly evolved genetic pathways or the modification of pre‐existing gene regulatory toolkits present in the charophyte ancestor (Langdale, 2008).

Among these numerous innovations, early land plants evolved structures that enabled the acquisition of nutrients in dry habitats. In *P. patens*, these structures are the FCC‐containing caulonemal filaments (reviewed in Ligrone et al., 2012). The evolution of 3D growth was another critical innovation in early land plants. 3D growth is an invariable feature of land plants, enabling increased plant productivity and diversity of form across the terrestrial biosphere (Harrison, 2017; Moody, 2020). Here we show that PpTPL is required for both the switch to 3D growth and caulonemal filament formation in the bryophyte *P. patens* (Figures 2 and 5).

Early land plant adaptions evolved in pro‐bryophytes and tracheophytes (Donoghue et al., 2021; Ligrone et al., 2012) and studies of extant bryophytes, such as *P. patens*, have expanded our understanding of the early stages of land plant evolution (Rensing et al., 2020). TPL represents a pre‐existing regulatory module that evolution has utilised to drive innovation. Given that TPL is essential for the formation of foraging tissues and 3D growth in *P. patens*, which were important for life on land, we conclude that TPL was an important facilitator of early terrestrial plant life.

It has recently been suggested that the charophyte ancestors were preadapted to land, and may have already been living on land before the embryophytes (Donoghue & Paps, 2020; Harholt et al., 2016). Since TPL predates both land plants and charophytes (Plant et al., 2021) it will be interesting to learn whether the role of this corepressor in phytoterrestrialisation is more ancient than the early land plants.

## EXPERIMENTAL PROCEDURES

### Plant growth

*Physcomitrium patens* ssp. *patens* (Hedwig) ecotype ‘Gransden 2004’ (Ashton & Cove, 1977; Rensing et al., 2008) was cultured on BCDAT medium at 25°C under continuous light as described previously (Knight et al., 2002; Nishiyama et al., 2000).

Arabidopsis wild‐type (Col‐0) and transgenic lines (in the Col‐0 background) were grown under long‐day conditions at 21°C, or at 27°C to enhance mutant phenotypes.

### *Physcomitrium patens* phenotypic analyses

For gross colony morphology analyses, small protonemal explants were inoculated onto BCDAT agar containing various additives as described in the Results section, and plant growth was monitored. For chloronemal and caulonemal filament development, small protonemal explants or spot inocula from homogenised protonema were plated onto BCDAT media with or without appropriate additives overlaid with cellophane discs. To examine production of caulonemata, small explants were inoculated onto BCD (BCDAT without ammonium tartrate) + 0.5% glucose (± various additives) plates overlaid with cellophane discs and incubated vertically in the dark at 21°C for 8 days (Hoffmann et al., 2014). Synchronised bud development was induced following a previously described protocol (Aoyama et al., 2012), with modifications suggested by N. Sugimoto, M. Hasebe and Y. Sato (National Institute for Basic Biology, Okazaki, Japan; personal communication), to include a final incubation step under continuous polarised white light. Structures were imaged using a Keyence VHX‐6000 digital microscope (Keyence, Osaka, Japan).

### Molecular biology

#### Generation of *PpTPL* gene‐targeting knockout constructs

For gene‐targeted knockout of *PpTPL1*, the 5′ and 3′ targeting sequences were PCR amplified using primer pairs TPL1_S1 + TPL1_A1 and TPL1_S2 + TPL1_A2, respectively. The 5′ and 3′ fragments were cloned on either side of the *hptIV* (Hygro^R^) cassette in a pMBL5‐derived plasmid (Kamisugi et al., 2005) to create vector pTPL1HKO. A 3383‐bp fragment was PCR amplified from pTPL1HKO using primers TPL1_KOS + TPL1_KOA for use in *P. patens* plant transformations.

For PpTPL2, the 5′ and 3′ targeting sequences were amplified using primers TPL2_S1 + TPL2_A1 and TPL2_S2 + TPL2_A2, respectively. The 5′ and 3′ fragments were cloned on either side of the *nptII* (G418^R^) selection cassette in a pMBL5‐derived plasmid (Kamisugi et al., 2005) to create vector pTPL2KO. A 3159‐bp fragment was PCR amplified from pTPL2KO using primer pair TPL2_KOS + TPL2_KOA for use in *P. patens* transformations.

A summary of the constructs, confirmation PCRs and primer positions is shown in Figure S8. All primer sequences and additional information are listed in Table S5.

#### Generation of PpTPL2 β‐estradiol‐inducible constructs

The *PpTPL2* coding sequence was amplified from wild‐type *P. patens* cDNA, using the Gateway modified primers PpT2‐F and PpT2‐R and cloned into the pDONR201 vector. The N176H mutation was generated with the Q5 site‐directed mutagenesis kit (New England Biolabs, Ipswich, MA, USA) and primers PpT2sdmF and PpT2sdmR. The N176H version of PpTPL2 was subcloned into pPGX8 (Kubo et al., 2013) to generate the pGX‐*PpTPL2* ^*N176H*^ plasmid. Plasmid DNA was linearised with *Pme*I for *P. patens* plant transformations and integration of the transgene into the *PIG1* genomic region (Okano et al., 2009). All primer sequences and additional information are listed in Table S5.

#### Generation of PpTPL β‐estradiol‐inducible RNAi constructs

For both *PpTPL1* and *PpTPL2*, gene synthesis was used to generate a DNA fragment that included approximately 350 bp inverted sequence repeats placed on either side of the GPA intron (Nakaoka et al., 2012) (see Figure S8). The synthesised fragments were cloned into pPGX8 to generate pGX‐*PpTPL1* ^*RNAi*^ and pGX‐*PpTPL2* ^*RNAi*^. Plasmids were linearised with *Pme*I for *P. patens* plant transformations.

#### Preparation of constructs for expression in Arabidopsis

The coding region of Arabidopsis *TPL* (At1g15750) was amplified from cDNA prepared from *tpl‐1* homozygous plants using primers AtTPL‐F and AtTPL‐R. The *tpl‐1* and *PpTPL2* ^*N176H*^ coding sequences were cloned into pALLIGATOR3 downstream of a constitutive 35S promoter (Bensmihen et al., 2004) to generate pALLIG3‐*tpl‐1* and pALLIG3‐*PpTPL2* ^*N176H*^ constructs. All primer sequences and additional information are listed in Table S5.

#### Quantitative real‐time polymerase chain reaction (qRT‐PCR)

Total RNA was extracted from approximately 100 mg of protonemal tissue (5 days post‐homogenisation) using the RNeasy Plant Mini Kit (QIAGEN, Hilden, Germany). cDNA was synthesised from 1 μg of total RNA using the iScript cDNA synthesis Kit (Bio‐Rad, Hercules, CA, USA). Quantitative real‐time polymerase chain reaction (qRT‐PCR) reactions were run in triplicate using a Bio‐Rad CFX96 Real‐Time System with SsoFast EvaGreen Supermix (Bio‐Rad). *PpTPL1* and *PpTPL2* transcript abundance was normalised to *PpEF1α* (Pp3c2_6770V3.1; Lloyd & Davies, 2013). Primers are listed in Table S5.

#### RNA sequencing

Homogenised protonemal tissue from wild‐type, *PpTPL2* ^*N176H*^ and *ΔPptpl2 PpTPL1* ^*RNAi*^ transgenic plants was inoculated onto BCDAT plates. After 5 days of growth, tissue was harvested, inoculated into 10 ml liquid BCDAT media ± 1 μm β‐estradiol and incubated for 8 h at 21°C under continuous light. 6‐Benzylaminopurine (BAP) was added to each culture at a final concentration of 100 nm to stimulate bud formation, and cultures were incubated for a further 40 h. Total RNA was prepared from approximately 100 mg tissue using the RNeasy Plant Mini Kit (QIAGEN). All steps were performed in duplicate or triplicate to ensure independent biological replicates for RNA sequencing (RNA‐seq). Oligo‐dT‐primed library preparation using the TruSeq sample preparation kit (Illumina, San Diego, CA, USA) and sequencing on an Illumina NextSeq platform to generate single‐end 75 nt long reads was performed by the University of Leeds Next Generation Sequencing Facility (dna2.leeds.ac.uk/genomics/index.php).

The quality of the raw reads was assessed using FastQC (www.bioinformatics.babraham.ac.uk/projects/fastqc/), Cutadapt (Martin, 2011) was used to trim adapter sequences, and fastq_quality_filter (hannonlab.cshl.edu/fastx_toolkit/) was used with the parameters ‐q 20 and ‐p 90. Subread aligner (Liao et al., 2013) was used to map the clean reads against the *P. patens* reference genome (Ensembl Plants release 40; Howe et al., 2020). BAM files containing only uniquely mapped reads were sorted and indexed using SAMtools (Li et al., 2009). The read counts were obtained using featureCounts (Liao et al., 2014) according to the GTF file associated with the reference genome file. DEGs were identified using DESeq2 (Love et al., 2014). FASTQ data were submitted to the Sequence Read Archive (www.ncbi.nlm.nih.gov/sra) under accession number PRJNA723997.

For comparison of the *ΔPptpl2 PpTPL1* ^*RNAi*^[+] data with control samples, RNA‐seq transcript abundances were quantified using Kallisto Quant (Bray et al., 2016) and DEGs were identified with DESeq2 (Love et al., 2014) on the Galaxy web platform (Afgan et al., 2018).

GO enrichment was examined using The Gene Ontology Resource (geneontology.org).

### Generation of transgenic plants

*Physcomitrium patens* transformations, DNA delivery, regeneration of protoplasts and selection of transformants on medium containing hygromycin or G418 were carried out using standard procedures (Kamisugi et al., 2005). All lines were screened by PCR (Ito et al., 2014; Kihara et al., 2006), as shown in Figure S8, using primers listed in Table S5.

To validate the dominant negative nature of *PpTPL2* ^*N176H*^ prior to transformation into *P. patens* plants, Arabidopsis plants containing *35S:PpTPL2* ^*N176H*^ or *35S:tpl‐1* constructs were generated using the floral dip method (Clough & Bent, 1998). Transgenic seed was selected using the GFP seed‐coat marker encoded by the pALLIGATOR3 vector. For *35S::PpTPL2* ^*N176H*^ and *35S:tpl‐1*, six and five independent primary lines were recovered, respectively. For each, two lines showing *tpl‐1*‐like phenotypes were selfed at 27°C to enhance *tpl‐1* seedling phenotypes, which were scored in the next generation.

### Total flavonoid content

*Physcomitrium patens* protonemal colonies were homogenised and grown on cellophane discs overlaid on BCDAT plates with or without estradiol for 7 days. Protonemal material was ground to a fine powder in liquid N_2_, resuspended in 80% (v/v) methanol to 250 μg ml^−1^ fresh weight and incubated at 60°C for 1 h. Extracts were cleared by centrifugation (1 min at 18 400 ***g***) and 5 μl of supernatant or serial dilutions of the quercetin standard (in 80% [v/v] methanol) were spotted onto a nylon filter and overlaid with 3 μl of 0.25% (w/v) diphenylboric acid 2‐aminoethylester (in 80% [v/v] methanol). Fluorescence was imaged using a G:BOX Chemi XX6 system (Syngene, Cambridge, UK) with GFP and autoexposure settings, and the mean grey value for each spot was measured using ImageJ. Background and autofluorescence were subtracted for each sample.

## Supporting information


**Figure S1.** *PpTPL1* and *PpTPL2* have overlapping expression patterns.

**Figure S2.** *Physcomitrium patens Pptpl* single mutant and Arabidopsis *35S:PpTPL2* ^*N176H*^ phenotypes.

**Figure S3.** *PpTPL* is required for the initiation of 3D growth.

**Figure S4.** Hierarchical clustering and principal component analysis of genes differentially expressed in *PpTPL2* ^*N176H*^[+] plants relative to controls.

**Figure S5.** Analysis of DEGs in *ΔPptpl2 PpTPL1* ^*RNAi*^[+] and comparison with those in *PpTPL2* ^*N176H*^[+].

**Figure S6.** Reduced *PpTPL* activity alters filament development.

**Figure S7.** Comparison of genes differentially expressed in *PpTPL2* ^*N176H*^[+] with those from the *aux/iaaΔ* null mutant or wild‐type plants treated with exogenous cytokinin.

**Figure S8.** Preparation of constructs used in this study.

**Table S1.** Summary of RNA‐seq data.

**Table S2.** DEGs between various WT and transgenic *PpTPL2* ^*N176H*^ samples, induced with estradiol (+) or mock‐treated (−).

**Table S3.** Expression of genes involved in 3D growth, the cell cycle, cell wall biogenesis, cytokinin metabolism and flavonoid biosynthesis is altered in *P. patens* plants with reduced TPL activity.

**Table S4.** Gene Ontology terms enriched among genes differentially regulated in *PpTPL2* ^*N176H*^[+] plants relative to controls.

**Table S5.** Oligonucleotides used during this study.
