# Supplementary figures and images for: The TOPLESS corepressor regulates developmental switches in the bryophyte Physcomitrium patens that were critical for plant terrestrialisation
Source: Plant J. 2023 Jun 8;115(5):1331–44. doi: 10.1111/tpj.16322 (PMC10953049; doi:10.1111/tpj.16322)

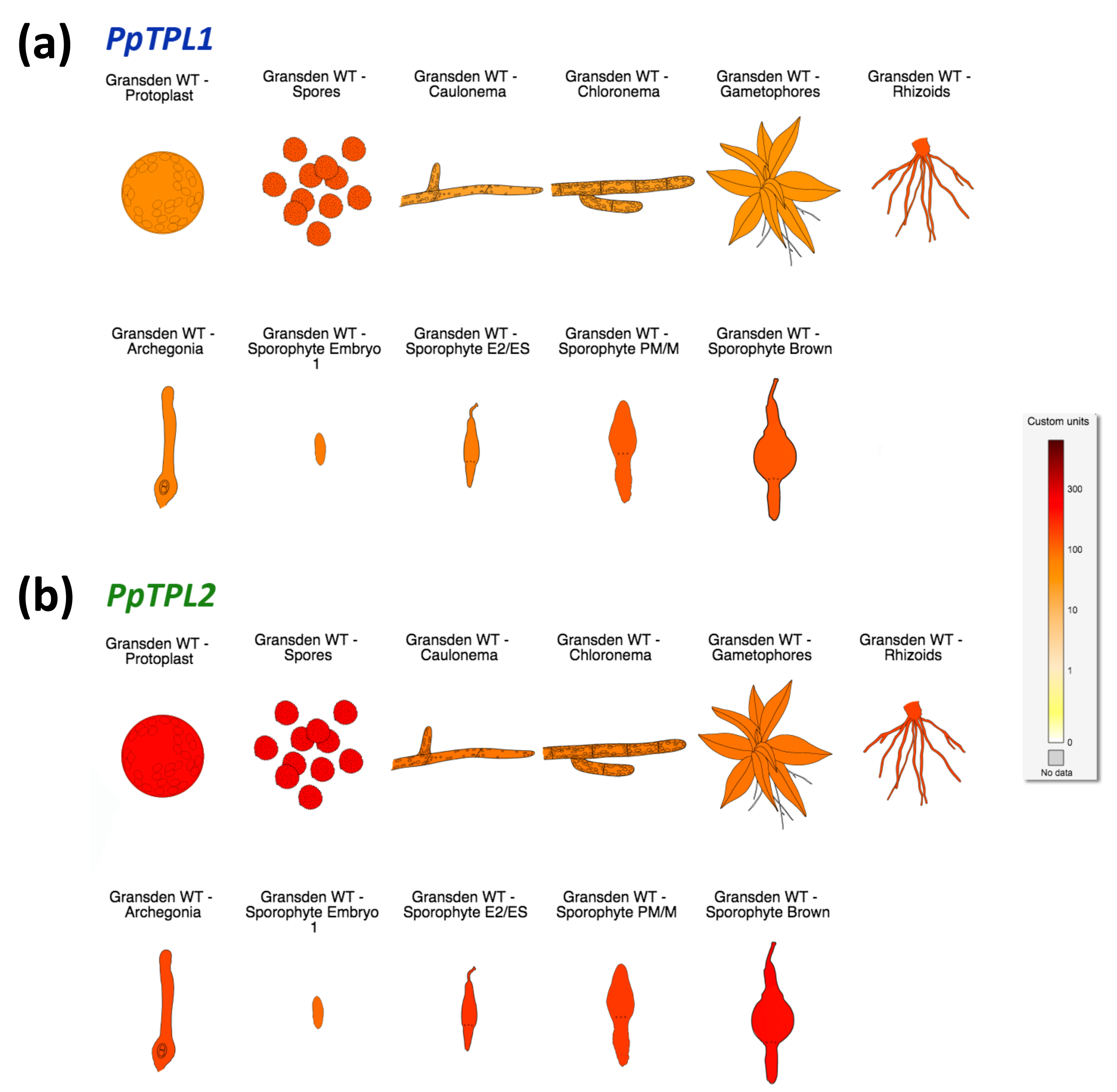

Supplement: Supplementary file 1 — Figure S1. PpTPL1 and PpTPL2 have overlapping expression patterns. [file TPJ-115-1331-s012.tif]

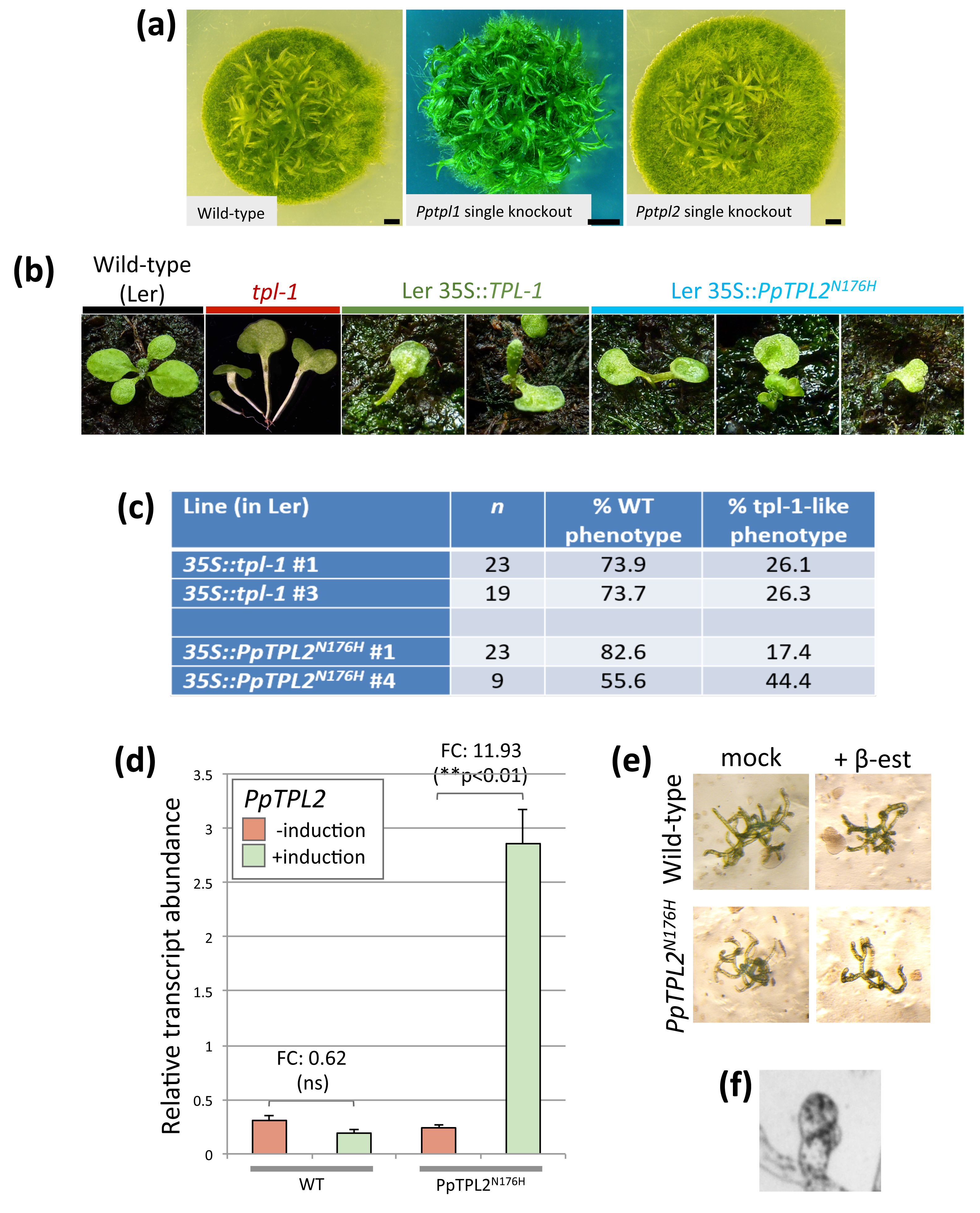

Supplement: Supplementary file 2 — Figure S2. Physcomitrium patens Pptpl single mutant and Arabidopsis 35S:PpTPL2 N176H phenotypes. [file TPJ-115-1331-s002.tif]

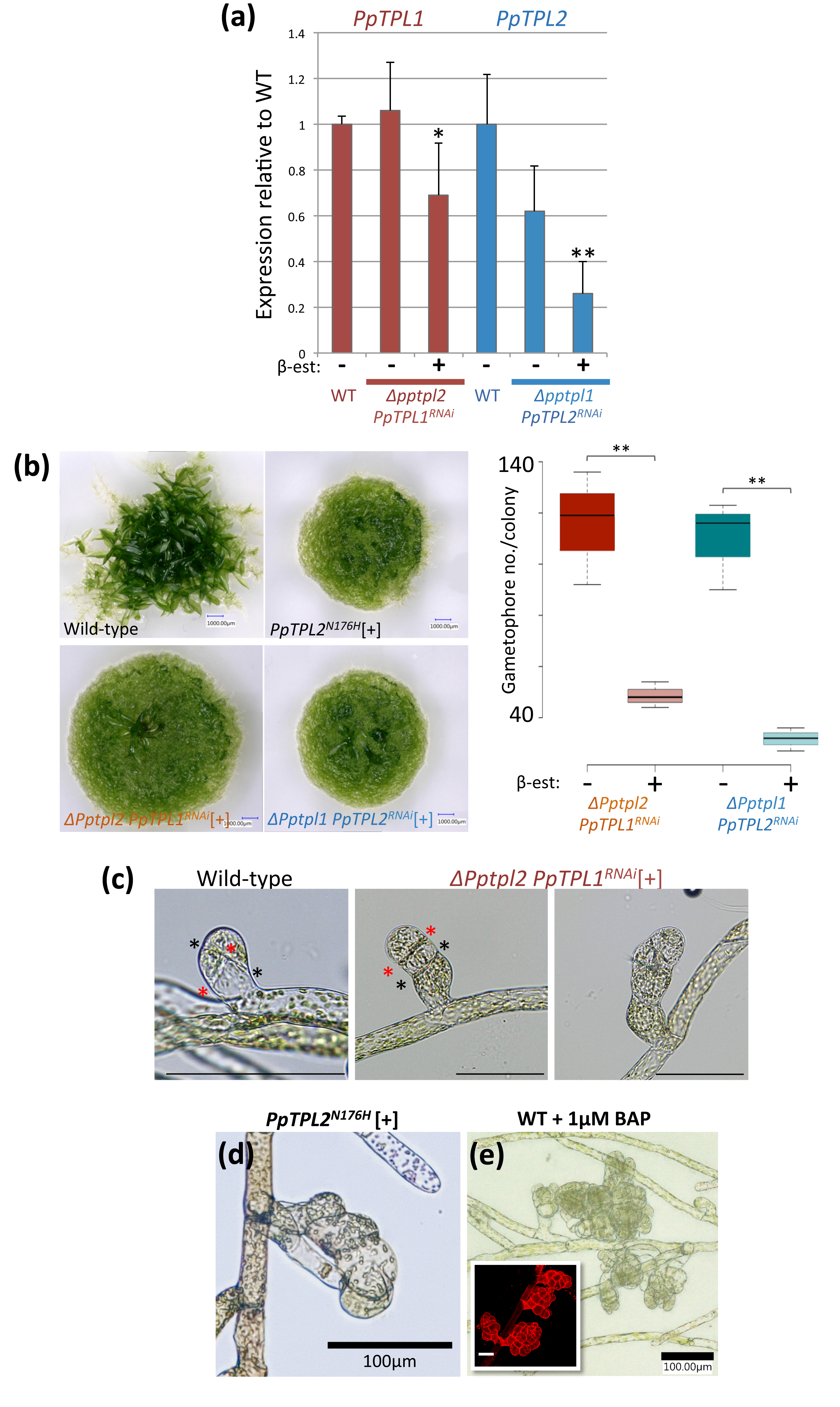

Supplement: Supplementary file 3 — Figure S3. PpTPL is required for the initiation of 3D growth. [file TPJ-115-1331-s013.tif]

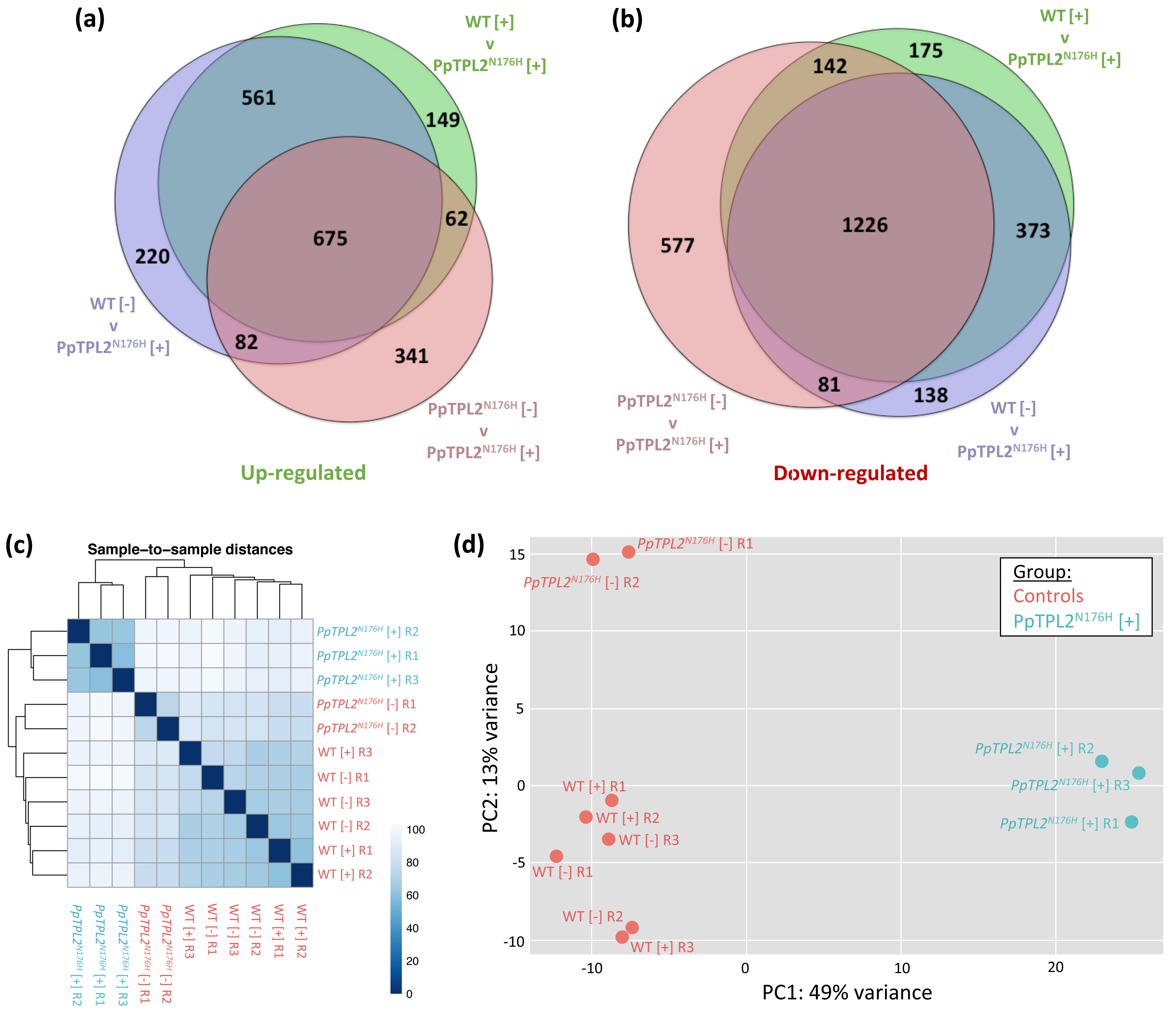

Supplement: Supplementary file 4 — Figure S4. Hierarchical clustering and principal component analysis of genes differentially expressed in PpTPL2 N176H [+] plants relative to controls. [file TPJ-115-1331-s003.tif]

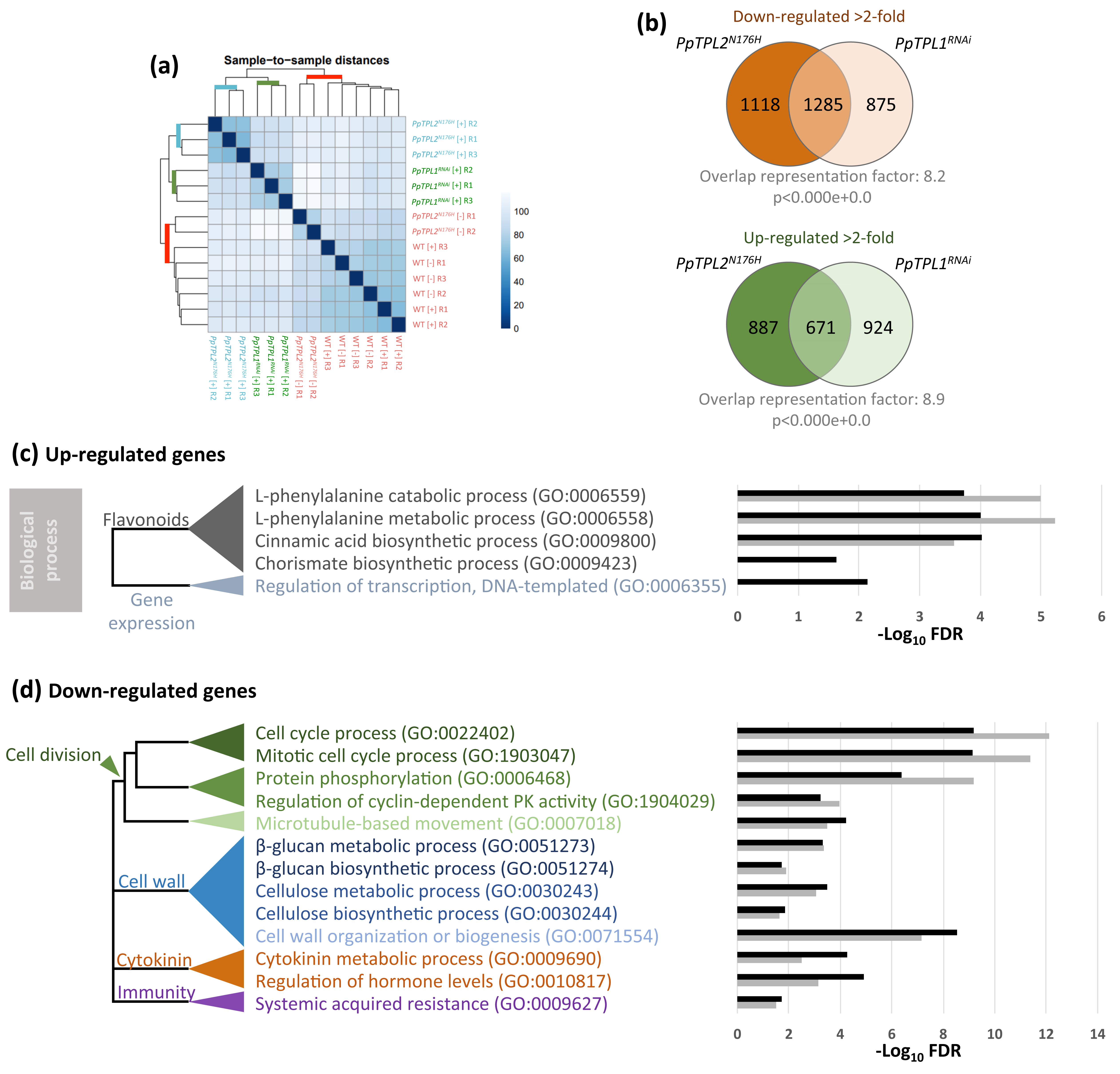

Supplement: Supplementary file 5 — Figure S5. Analysis of DEGs in ΔPptpl2 PpTPL1 RNAi [+] and comparison with those in PpTPL2 N176H [+]. [file TPJ-115-1331-s007.tif]

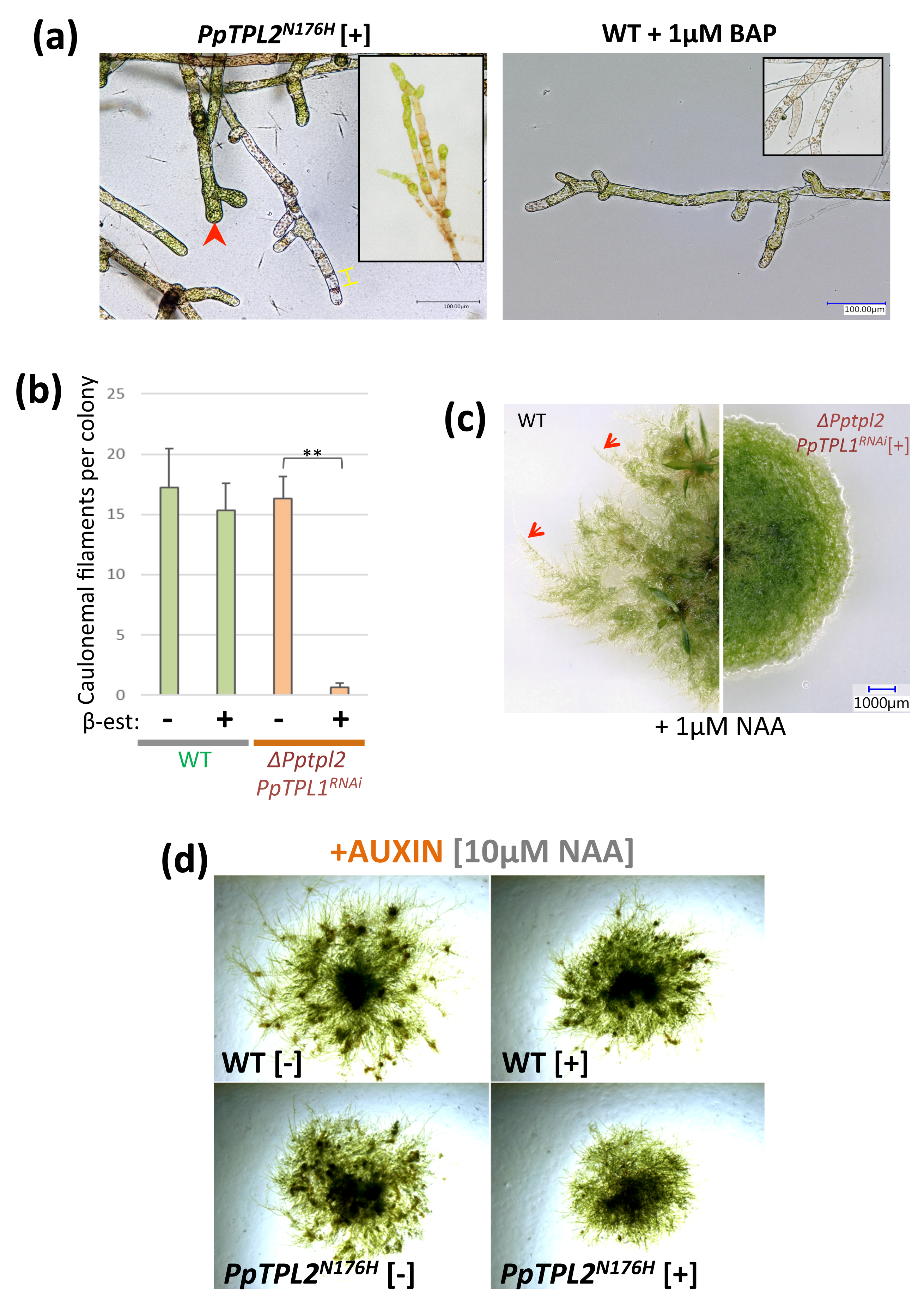

Supplement: Supplementary file 6 — Figure S6. Reduced PpTPL activity alters filament development. [file TPJ-115-1331-s010.tif]

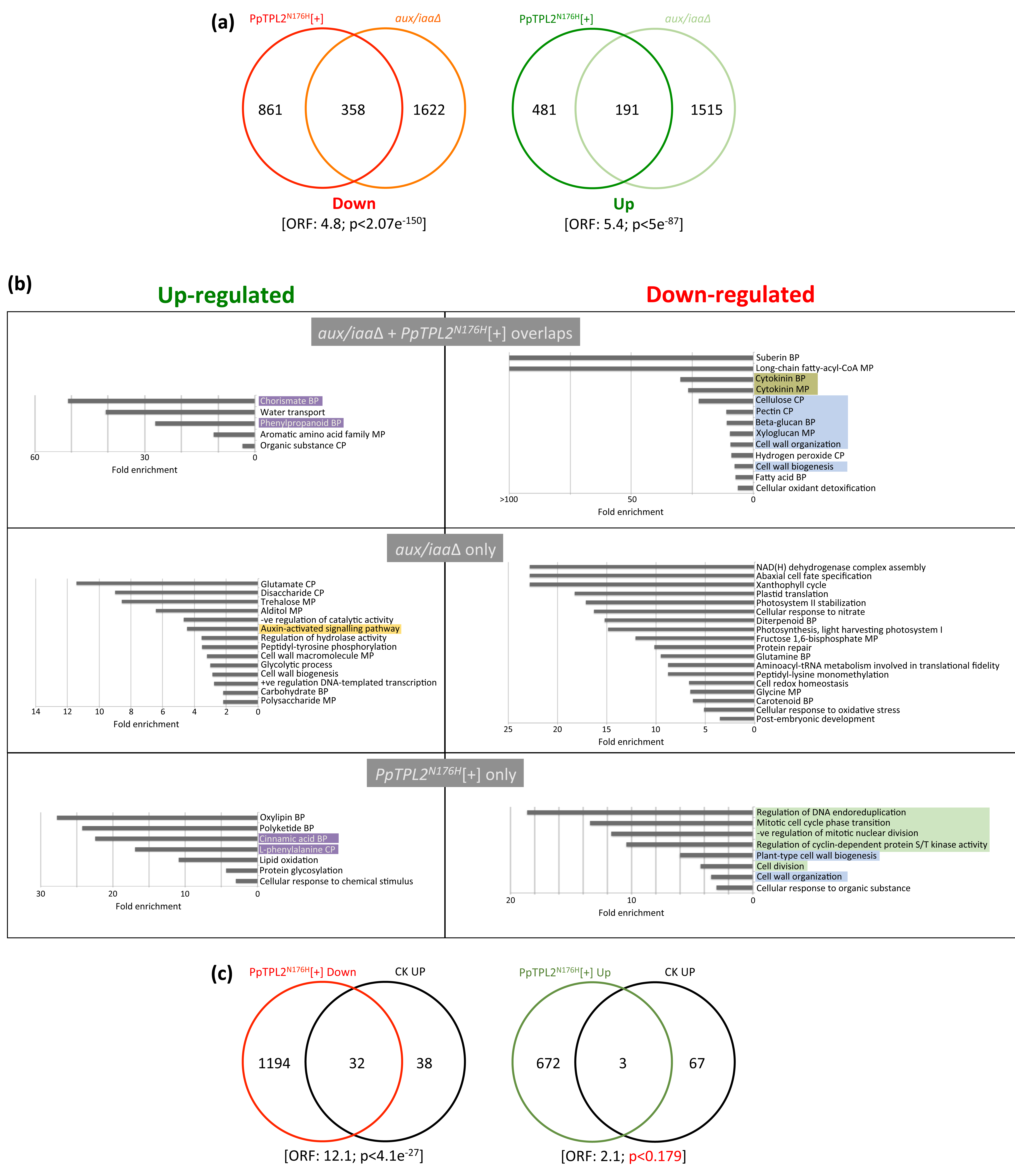

Supplement: Supplementary file 7 — Figure S7. Comparison of genes differentially expressed in PpTPL2 N176H [+] with those from the aux/iaaΔ null mutant or wild‐type plants treated with exogenous cytokinin. [file TPJ-115-1331-s005.tif]
